# Disaster: Are We Prepared?

**Published:** 2014-06-15

**Authors:** Negin Masoudi Alavi

**Affiliations:** 1 Trauma Nursing Research Center, Kashan University of Medical Sciences, Kashan, IR Iran

**Keywords:** Disasters, Nursing, Civil Defense

In the last issue of Nursing and Midwifery Studies Journal, there was an interesting article about the experiences of nurses in Bam earthquakes in December 2003 that left 25000 dead and put the whole nation into the shock and grief (1). In summary, nurses reported that there was such a mental stress that many of them could not perform their usual simple tasks efficiently. There was a shortage of essential equipment and weak management that led to chaos. As a whole, the health system and nurses were not prepared for that disaster (1). The question is “are we prepared for the next disaster?” Iran is the tenth disaster-prone country in the world and the fourth in Asia (2) and unfortunately, the next disaster will happen sooner or later. Large-scale disaster events such as earthquake, causes social and psychological distortion of the community, infrastructures, and service levels; in this situation, delivering the best healthcare services is a challenging task (3). Therefore, the health system especially nurses should be completely prepared for such events. Although some national efforts have been made, we cannot see the complete preparedness in our health system, as in the recent earthquake in Azerbaijan, the relief operation was far from satisfactory level. There are some lessons that we can learn from our past as well as from the experiences in other countries:

1. Hospitals can act as a source of support and management during disasters. The hospital disaster resilience, especially the focus of community-wide disaster cooperation, on-site medical rescue, and hospital patient care capacity must be improved. Hospitals need to take a more cohesive approach to be resilient and to cope efficiently in a potential disaster (4).
2. Nurses should be equipped through education and training with the necessary knowledge and abilities to work in a disaster. The International Council of Nurses (ICN) framework of nursing disaster competencies for general nurses can be used as a guideline for nursing preparedness (4). The emergency drills are essential part of nursing preparedness. The especial well-trained and well-equipped nursing teams should be available to move to the scene in the first hours after disaster.
3. There should be written disaster-specific plans for different situations such as pandemic influenza, earthquakes, floods, and landslides (5).
4. Nongovernmental organizations (NGOs) should be developed to organize the volunteers during the disasters. In Bam earthquake, the crowds of volunteers who rushed to offer help caused a lot of chaos and along with weak management, led to the misuse of human resources (1).

As a largest healthcare workgroup, nurses will be called upon during disasters to provide aid and care utilizing their unique skills, abilities, and understanding of the community. Fortunately there are a huge scientific and research-based materials concerning disaster, its management, and nursing in disaster that can be used for the preparedness.

To be effective, nurses must be prepared; this preparation has different dimensions including education and training, leadership, and having disaster-specific plans. The health organizations should take essential steps to manage future disasters more efficiently.
